# Insulin Resistance Indices and Cardiovascular Disease Risk in Middle-Aged and Older Adults with Prediabetes: A Machine Learning-Assisted Predictive Modeling Study

**DOI:** 10.3390/healthcare14162617

**Published:** 2026-08-19

**Authors:** Ya-Jie Zhai, Xiao-Yu Ding, Xin-Zhong Zhang, Xiao-Ying Ren, Guang Wang, Jia Liu

**Affiliations:** Department of Endocrinology, Beijing Chao-Yang Hospital, Capital Medical University, No. 8, Gongti South Road, Chaoyang District, Beijing 100020, China; zhaiyajie@mail.ccmu.edu.cn (Y.-J.Z.); dxycy0828@163.com (X.-Y.D.); zhangxinzhong0901@mail.ccmu.edu.cn (X.-Z.Z.); rxy178323779@mail.ccmu.edu.cn (X.-Y.R.)

**Keywords:** aging, prediabetes, triglyceride–glucose, cardiovascular disease, C-reactive protein–triglyceride–glucose index, weight-adjusted waist index

## Abstract

**Highlights:**

**What are the main findings?**

- CTI and TyG mediate 22–26% of the WWI–CVD association.
- CTI, TyG, and TyG-WWI are independently associated with CVD in prediabetes.

**What is the implication of the main finding?**

- Central obesity and insulin resistance should be assessed together for better CVD risk stratification in prediabetes.

**Abstract:**

**Background:** Insulin resistance (IR) indices—C-reactive protein–triglyceride–glucose index (CTI), triglyceride–glucose (TyG) index, and weight-adjusted waist index (WWI)—are linked to cardiovascular disease (CVD), but their comparative utility in prediabetic populations remains unclear. To evaluate the associations and exploratory mediating roles of CTI, TyG and WWI with prevalent CVD in middle-aged and elderly patients with prediabetes. **Methods:** The study included 3121 NHANES participants aged ≥45 with prediabetes. Four machine learning algorithms identified key variables. Multivariable logistic regression and restricted cubic spline analyses examined the associations between IR and CVD. Model performance was assessed via receiver operating characteristic curves, net reclassification improvement, integrated discrimination improvement, calibration, and decision curve analysis. Subgroup analyses tested robustness. Mediation analysis assessed the mediating roles of IR in the WWI–CVD relationship. External validation used CHARLS. **Results:** Individuals in the highest tertiles of TyG, TyG–WWI, and CTI had approximately twofold higher CVD risk (ORs: 1.80–1.97). RCS showed a significant linear increase in CVD risk with higher IR index levels. In fully adjusted models, AUCs improved markedly over baseline: TyG–WWI 0.771 vs. 0.579. Mediation analyses indicated that CTI and TyG mediated 22.13–26.30% of the WWI–CVD association. Subgroup analyses and external validation in CHARLS supported the robustness. **Conclusions:** IR, as captured by TyG, TyG–WWI, and CTI, independently elevates CVD risk among middle-aged and older adults with prediabetes. WWI is associated with greater odds of prevalent CVD, potentially driven by adiposity accumulation, with CTI and TyG partially mediating this effect.

## 1. Introduction

Type 2 diabetes mellitus (T2DM) is a chronic metabolic disorder characterized by dysregulation of glucose and lipid metabolism. The stage preceding the onset of diabetes, marked by elevated blood glucose levels below the diagnostic threshold for diabetes, is defined as prediabetes [1]. According to global estimates, in addition to 589 million people living with diabetes worldwide, approximately 635 million individuals are affected by prediabetes as of 2025 [2]. China bears the greatest diabetes burden globally, with about 148 million diabetic adults [2]. Cardiovascular diseases (CVD), encompassing a range of cardiac and vascular disorders, represent a leading cause of mortality and disability worldwide and pose a major public health challenge [3]. In patients with T2DM, the coexistence of insulin resistance (IR), hyperglycemia, dyslipidemia, abdominal obesity, and a prothrombotic state contributes to an elevated risk of CVD [4]. A meta-analysis indicated that individuals with prediabetes have a 13–30% higher risk of developing CVD compared with those with normal glucose levels [5]. Moreover, CVD-related complications—such as impaired cardiac function, reduced work capacity, and the need for long-term pharmacological treatment—impose a substantial economic burden on both society and families [6,7]. The pathophysiological mechanisms linking diabetes to CVD involve multiple interrelated processes, including disruptions in cardiac energy metabolism, glucolipotoxicity, mitochondrial dysfunction, oxidative stress, and chronic inflammation. These mechanisms collectively contribute to structural and functional myocardial injury, ventricular remodeling, and ultimately, the progression of CVD [8,9,10].

Traditional markers such as fasting blood glucose (FBG) alone cannot capture the complex lipid metabolism disorders in prediabetes. The triglyceride–glucose (TyG) index has demonstrated superior diagnostic performance compared with the homeostasis model assessment of insulin resistance in predicting metabolic syndrome and nonalcoholic fatty liver disease [11,12]. Therefore, the C-reactive protein–triglyceride–glucose index (CTI), first introduced by Ruan et al., is a novel composite marker that integrates inflammatory and metabolic components, enabling a concurrent assessment of systemic inflammatory status and the severity of IR [13]. Obesity and IR are well-established major risk factors for the development of diabetes [14]. The weight-adjusted waist index (WWI) is a recently developed obesity indicator that reflects central obesity independent of body weight, and it has been shown to outperform conventional anthropometric measures in predicting the risk of metabolic disorders [15,16]. TyG has demonstrated strong diagnostic performance in identifying IR [17]. Accumulating evidence indicates that IR is not only positively associated with the risk of prediabetes [18] but also plays a critical role in the onset and progression of CVD among diabetic patients [19]. Combining TyG with WWI may offer superior integrated value for assessing IR and related metabolic abnormalities [20]. Therefore, early identification and intervention of IR could represent a promising strategy for the prevention of CVD [19,21].

Previous studies have established a clear association between TyG and the risk of cardiovascular events [22,23]. However, evidence remains scarce regarding the comparative discriminative ability of CTI, TyG, and its derivative TyG-WWI—particularly during the critical prediabetes stage. To address this gap, we applied four machine learning algorithms to identify key confounding variables and comprehensively evaluate the associations of CTI, TyG, and TyG-WWI with prevalent CVD. We systematically examined the incremental discriminative value of these indicators beyond traditional baseline models and explored the potential mediating roles of CTI and TyG in the relationship between WWI and prevalent CVD. This study aims to provide novel evidence for early risk stratification and precision prevention of CVD.

## 2. Methods

### 2.1. Data Sources

This cross-sectional study utilized data from the National Health and Nutrition Examination Survey (NHANES) as the primary discovery cohort. Conducted by the National Center for Health Statistics (NCHS) under the Centers for Disease Control and Prevention, NHANES employs a complex, stratified, multistage probability sampling design to collect comprehensive data on demographic characteristics, socioeconomic status, dietary intake, physical examinations, and laboratory measurements. The survey protocol was approved by the NCHS Research Ethics Review Board.

For external validation, data were obtained from the China Health and Retirement Longitudinal Study (CHARLS), a nationally representative longitudinal survey targeting the Chinese population aged 45 years and older [24]. CHARLS covers multidimensional information, including sociodemographic characteristics, economic status, and health-related behaviors. The study was approved by the Peking University Institutional Review Board (IRB No. IRB00001052–11015). All participants in both surveys provided informed consent prior to their inclusion.

### 2.2. Inclusion and Exclusion Criteria

This study utilized data from NHANES (1999–2010), which initially included 3306 participants aged 45 years or older. All included individuals met the diagnostic criteria for prediabetes, defined by at least one of the following conditions: fasting plasma glucose between 100–125 mg/dL (5.6–6.9 mmol/L), glycated hemoglobin (HbA1c) between 5.7–6.4%, or 2 h oral glucose tolerance test plasma glucose between 7.8–11.0 mmol/L (indicating impaired glucose tolerance).

Participants were excluded based on the following criteria: (1) missing key demographic or anthropometric data (age, sex, height, weight, waist circumference); (2) missing essential laboratory measurements (fasting glucose, HbA1c, C-reactive protein, triglycerides); (3) zero sample weight; (4) incomplete questionnaire information regarding CVD, including congestive heart failure, coronary heart disease, angina pectoris, myocardial infarction, or stroke. Finally, 3121 participants from the NHANES database were included in the final analysis (as shown in Figure S1). Additionally, data from the CHARLS 2011 wave were incorporated for subsequent external validation. Detailed information regarding the CHARLS cohort is provided in Supplementary Material S1.

### 2.3. Calculation of CTI, TyG and TyG-WWI

In this study, CTI, TyG and TyG-WWI were used to evaluate the level of IR. The specific calculation formula is as follows:

$$WWI=WC\left(cm\right)/\sqrt{Weight(kg)};$$

$$TyG=\ln\left[TG\left(mg/dl\right)\times fasting\;glucose\left(mg/dl\right)/2\right];$$

TyG-WWI=TyG×WWI;

$$CTI=0.412\times \ln\left(CRP\left[mg/L\right]\right)+\ln\left(TG\left[mg/dl\right]\times FBG\left[mg/dl\right]\right)/2.$$

### 2.4. Assessment of CVD

CVD was ascertained through a self-reported questionnaire. Participants were asked whether a physician or other health professional had ever informed them of having any of the following conditions: congestive heart failure, coronary heart disease, angina pectoris, myocardial infarction, or stroke. In the NHANES database, this information was derived from the Medical Conditions Questionnaire (items MCQ160b–f). Participants who responded “yes” to any of these conditions were classified as having a positive CVD history [23].

### 2.5. Covariates

This study is based on the NHANES database. The covariates included in the system include socio-demographic factors: gender (male, female); age; education level (below high school, high school/GED, college or above) and marital status. Lifestyle factors: smoking status (current smokers, ex-smokers, never smokers) and alcohol consumption (no, 1–5 cups/month, 6–10 cups/month, >10 cups/month). Body measurements include weight, height, waist circumference (WC), blood pressure (systolic blood pressure, diastolic blood pressure) [25]. Body mass index (BMI) was classified according to the National Institutes of Health criteria: underweight (<18.5 kg/m^2^), normal (18.5–24.9 kg/m^2^), overweight (25.0–29.9 kg/m^2^) and obese (≥30 kg/m^2^). Abdominal obesity is defined as WC ≥ 102 cm in men and ≥88 cm in women [26]. Hypertension (diagnosed or average systolic blood pressure ≥ 140 mmHg or diastolic blood pressure ≥ 90 mmHg); laboratory indicators: FBG, HbA1c, triglyceride (TG), total cholesterol (TC), uric acid (UA), serum creatinine (Cr), blood urea nitrogen (BUN) and C-reactive protein (CRP) were collected. CRP was detected by latex-enhanced turbidimetry. For variables with missing data, the missing rate of all variables is less than 5%. The MICE package in R 4.5.1 is used for multiple imputation (m = 5), and techniques such as predictive mean matching and logical rule-based imputation are comprehensively used to reduce selective bias and maintain sample representativeness.

### 2.6. Statistical Analysis

The distribution of continuous variables was evaluated by Q-Q plot and Kolmogorov–Smirnov test. The normal distribution variables are expressed as mean and standard deviation (SD), and the skewed variables are expressed as median and interquartile range (IQR). The *t*-test or Mann–Whitney U test for complex survey samples is used for comparison of continuous variables. The categorical variables were described by weighted counts and percentages, and the differences between groups were evaluated by the Rao–Scott-corrected chi-square test.

To control for potential confounders while optimizing model predictive performance, four machine learning algorithms were employed to screen key predictor variables (See Supplementary Material S1 for specific methods): Support Vector Machine–Recursive Feature Elimination (SVM-RFE) was used to identify features with nonlinear discriminative boundaries; Least Absolute Shrinkage and Selection Operator (LASSO) regression applied L1 penalty to achieve variable sparsity and selection; the Boruta algorithm, based on random forest and shadow variable comparison, assessed the true importance of features; and Extreme Gradient Boosting (XGBoost) quantified the gain contribution of each variable within the gradient boosting framework. Each algorithm selected the top 10 predictors most strongly associated with CVD based on its internal importance metrics. The final covariate set was constructed by taking the union of all unique variables selected by at least one algorithm, with priority given to variables selected by multiple methods (see Supplementary Table S1). After integration and deduplication, this consolidated set was used for covariate adjustment in Model 3.

**Figure 1:**
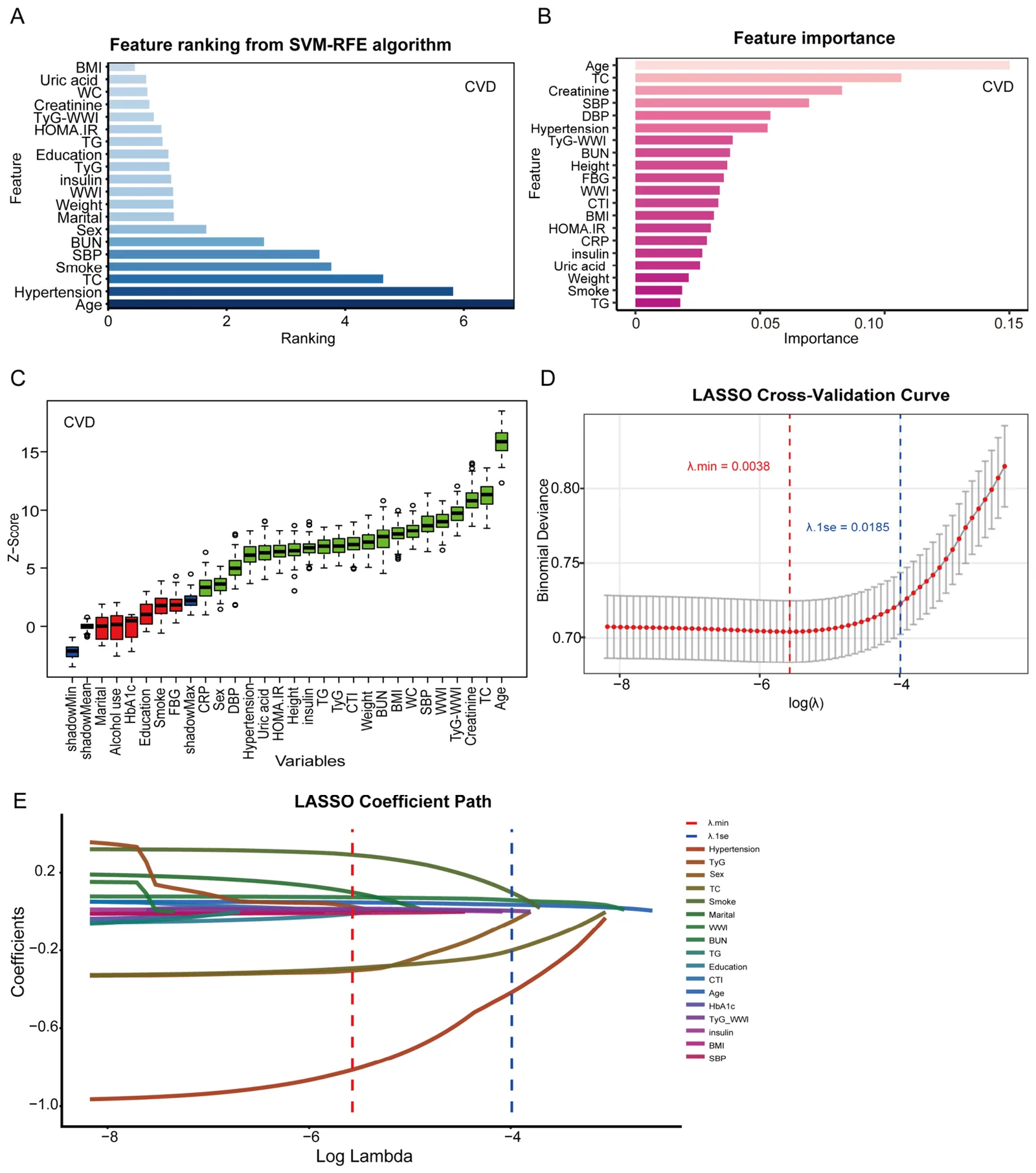
Machine learning-based feature selection for insulin resistance indices (CTI, TyG, TyG-WWI) in relation to CVD. Four algorithms independently rank candidate covariates; the top 10 variables per algorithm are integrated into the final covariate set. (**A**) SVM-RFE: variables retained after successive elimination of the least-informative 20 features; later removal indicates higher importance. (**B**) XGBoost: top 20 predictors ranked by standardized gain; lower rank denotes greater contribution to model performance. (**C**) Boruta: importance confirmed over 500 permutation runs; horizontal axis, input variables; vertical axis, Z-score significance (green, accepted; red, rejected). (**D**) LASSO ten-fold cross-validation: binomial deviance versus log(λ); upper x-axis, number of included variables; dashed lines mark λmin and λ1se. (**E**) LASSO coefficient path: shrinkage trajectories of individual predictors across log(λ); CVD, cardiovascular disease; BMI, body mass index; SBP, systolic blood pressure; DBP, diastolic blood pressure; FBG, fasting blood glucose; HbA1c, hemoglobin A1c; BUN, blood urea nitrogen; CRP, C-reactive protein; WC, waist circumference; WWI, weight-adjusted waist index; TyG, triglyceride–glucose index; CTI, C-reactive protein–triglyceride–glucose index.

To evaluate the independent association between novel IR indices (CTI, TyG, TyG-WWI) and CVD risk, binary logistic regression was performed. Each index was divided into tertiles, with the lowest tertile (T1) as the reference group. Odds ratios (ORs) and 95% confidence intervals (CIs) for CVD were calculated across three progressively adjusted models: Model 1 was unadjusted; Model 2 adjusted for demographic variables including age, sex, educational level, and marital status; and Model 3 further incorporated the subset of top 10 variables identified through the four machine learning feature selection methods. Results are visualized in Figure 1. In order to evaluate the multicollinearity between variables, the generalized variance inflation factor was calculated. In order to verify the robustness of the results, the use of antihypertensive drugs, lipid-lowering drugs and hypoglycemic drugs was additionally corrected on the basis of Model 3, and sensitivity analysis was carried out.

To explore potential nonlinear relationships between the IR indices and CVD, restricted cubic splines (RCS) were fitted using the rms package in R. Smooth curves were generated based on generalized linear models, with the number and position of knots determined by the Akaike Information Criterion (AIC) in conjunction with variable distribution characteristics, thereby balancing model fit and complexity. Adjustments for potential confounders were consistent with those in the logistic regression models.

To evaluate the predictive performance of the three IR indices for CVD risk, receiver operating characteristic (ROC) curves were plotted, and the area under the curve (AUC) along with the C-statistic were computed to assess discriminative ability. The net reclassification improvement (NRI) and integrated discrimination improvement (IDI) were calculated to quantify improvements in risk stratification accuracy in the fully adjusted model. Calibration curves were used to evaluate the agreement between predicted probabilities and observed event rates. Calibration performance was quantitatively assessed using calibration slope, calibration intercept, and Brier score. Decision curve analysis (DCA) was applied to assess the net clinical benefit of the models. Furthermore, the predictive performance of the three indices was compared via ROC analysis, and the DeLong test was used to evaluate statistical differences in AUC between models. To further assess the incremental predictive value of TyG-WWI beyond established clinical predictors, a base clinical model was constructed incorporating age, sex, systolic blood pressure, diastolic blood pressure, smoking status, TC, HDL-C, and HbA1c—variables encompassing the core components of standard risk scores. Three nested models were compared: (1) the base clinical model alone; (2) the base clinical model plus TyG; and (3) the base clinical model plus TyG-WWI.

To clarify the potential influence of factors that may affect the relationship between IR indices and CVD, multiple sensitivity analyses were conducted. Subgroup analyses were performed based on age (<60, ≥60 years), sex (male, female), smoking status (ever, never), alcohol use (yes, no), and BMI (<25, ≥25 kg/m^2^). Within each stratum, logistic regression models adjusted for the same set of covariates as in the main analysis were used to assess the association between IR indices and CVD. Interaction terms were introduced to test for effect modification between stratification factors and the indices on CVD risk.

To further investigate whether WWI may explain the underlying pathogenesis and potential cascading effects in the relationship between CVD and indicators of IR and inflammation, we conducted a mediation analysis to evaluate the potential mediating roles of novel IR indicators (CTI and TyG) in the association between WWI and CVD. Based on binary logistic regression models, mediation analysis was performed using the “mediation” package, and the robustness of the results was assessed using bootstrap resampling with 1000 replications. Through this approach, the total effect of WWI on CVD was decomposed into its direct effect and the indirect (mediated) effects through CTI and TyG, with mediation proportions calculated and statistical significance tested.

In the CHARLS cohort, baseline characteristics were analyzed using standardized methods consistent with those applied in the NHANES cohort. To comprehensively validate the predictive performance of the three IR indices, we evaluated discrimination (AUC, DeLong test), calibration (calibration slope, calibration intercept), and overall accuracy (Brier score) in the fully adjusted model, in addition to prevalence trends across tertiles. Participants were categorized into three subgroups according to tertiles of CTI, TyG, and TyG-WWI. CVD prevalence was calculated for each subgroup, and the Cochran–Armitage trend test, along with logistic regression models treating the tertiles as a continuous variable, were used to analyze trends in CVD prevalence with increasing levels of IR.

According to the NHANES analysis guidelines, this study used weight merging and weighting methods in the corresponding statistical models to consider complex sampling designs [27]. All analyses were performed using R software (version 4.5.1). Statistical tests were performed using a two-sided test. *p* < 0.05 was considered statistically significant.

## 3. Results

### 3.1. Clinical Baseline Characteristics

Table 1 summarizes the baseline clinical characteristics of the 3121 participants after exclusion and multiple imputation, among whom 441 had CVD. Regarding sociodemographic features, participants in the CVD group were significantly older, more likely to be male, less frequently married, and had lower educational attainment compared with those in the non-CVD group (all *p* < 0.05). In terms of lifestyle and clinical profiles, the CVD group had more current smokers and hypertensive patients (all *p* < 0.05). No significant differences were observed between the two groups in terms of current alcohol consumption or abdominal obesity (all *p* > 0.05). With respect to anthropometric and metabolic parameters, the CVD group exhibited significantly lower diastolic blood pressure (DBP) and TC levels, along with significantly higher levels of UA, TG, Cr, BUN, and WWI (all *p* < 0.05). However, no significant differences were detected between the groups in terms of weight, height, BMI, WC, systolic blood pressure (SBP), FBG, HbA1c, or CRP (all *p* > 0.05). Notably, all three novel IR indices—TyG, TyG-WWI, and CTI—were significantly elevated in the CVD group (all *p* < 0.05).

### 3.2. Feature Selection and Independent Association of IR Indices with CVD

Multivariable-adjusted binary logistic regression was employed to investigate the independent associations between three novel IR indices—CTI, TyG, and TyG-WWI—and the risk of CVD in middle-aged and older adults with prediabetes (Table 2). Three progressively adjusted models were constructed: Model 1 was unadjusted; Model 2 incorporated basic demographic variables; and Model 3, serving as the primary analytical model, further included covariates selected through an integrated machine learning approach (SVM-RFE, XGBoost, Boruta, and LASSO regression). The variable selection process is illustrated in Figure 1 and detailed in Supplementary Table S1. Each IR index was categorized into tertiles, with T1 used as the reference. In the unadjusted model (Model 1), the highest tertile (T3) of CTI, TyG, and TyG-WWI was significantly associated with an increased risk of CVD (all *p* < 0.05). This association remained statistically significant after adjusting for demographic variables in Model 2 (all *p* < 0.05). Further adjustment for machine learning-selected covariates in Model 3 maintained the significant positive association between the highest tertile of all three indices and CVD risk (all *p* < 0.05), with a statistically significant linear trend observed across tertiles. Specifically, in the fully adjusted Model 3, participants in the highest tertile of CTI (OR = 1.80; 95% CI: 1.20–2.71), TyG (OR = 1.95; 95% CI: 1.23–3.09), and TyG-WWI (OR = 1.97; 95% CI: 1.20–3.23) had 1.80- to 1.97-fold higher odds of CVD compared with those in the lowest tertile. Sensitivity analysis showed that the above correlation remained significant (Supplementary Table S2) after additional correction of related drugs, confirming the robustness of the results.

### 3.3. Nonlinear Association Between Novel IR Indices and CVD Risk

**Figure 2:**
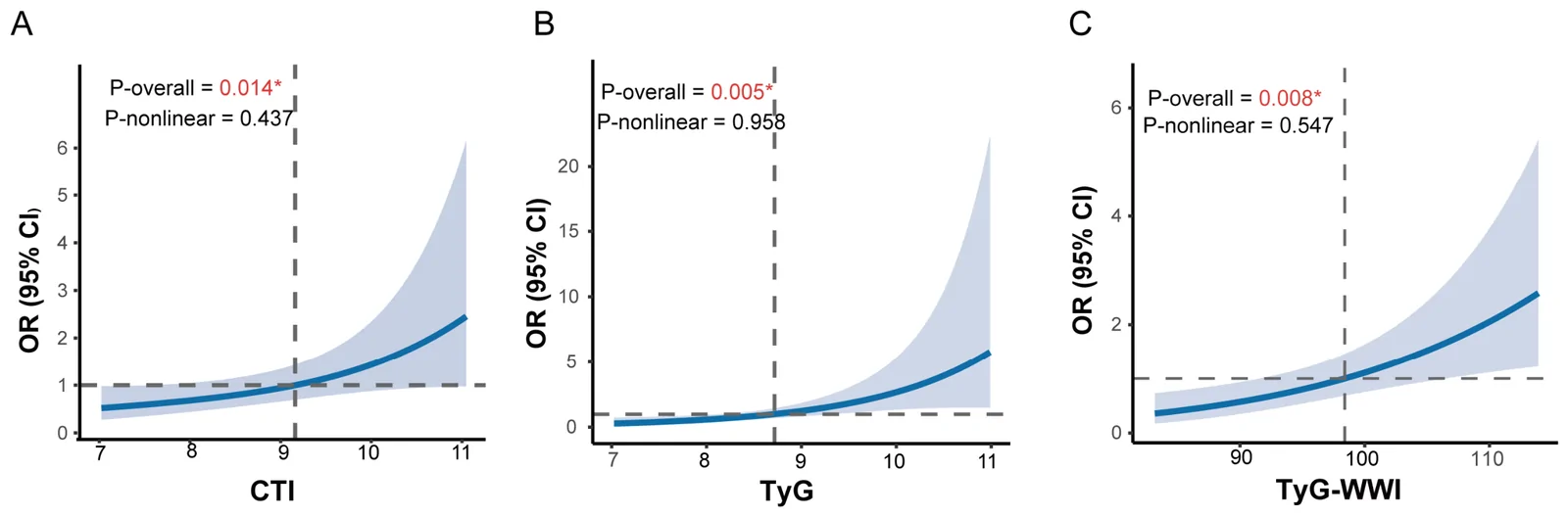
Nonlinear analysis of insulin resistance-related indices (CTI, TyG, TyG-WWI) and the risk of cardiovascular disease in prediabetes patients. Nonlinear associations between CTI, TyG, TyG-WWI and cardiovascular disease in middle-aged and elderly prediabetes patients were explored using restricted cubic spline (RCS) models and fitted with smooth curves. The three new insulin resistance-related indicators were (**A**) C-reactive protein–triglyceride–glucose index (CTI), (**B**) triglyceride–glucose index (TyG), and (**C**) Triglyceride–glucose weight-adjusted waist index (TyG-WWI). * *p* < 0.05.

RCS models were applied to examine potential nonlinear relationships between the novel IR indices (CTI, TyG, and TyG-WWI) and CVD risk in middle-aged and older adults with prediabetes. The models were adjusted for demographic characteristics and key covariates identified through multiple machine learning algorithms, as illustrated in Figure 2. Consistent with the logistic regression results, the RCS-based dose–response relationships revealed significant positive associations between all three IR indices and CVD risk. Specifically, elevated levels of CTI, TyG, and TyG-WWI were associated with a progressively increasing risk of CVD, demonstrating a significant linear trend. No evidence of nonlinearity was detected for any of the indices (all *p* for overall association <0.05; all *p* for nonlinearity >0.05).

### 3.4. Comprehensive Validation and Comparison of Novel IR Indices in Predicting CVD Risk Among Middle-Aged and Older Adults with Prediabetes

**Figure 3:**
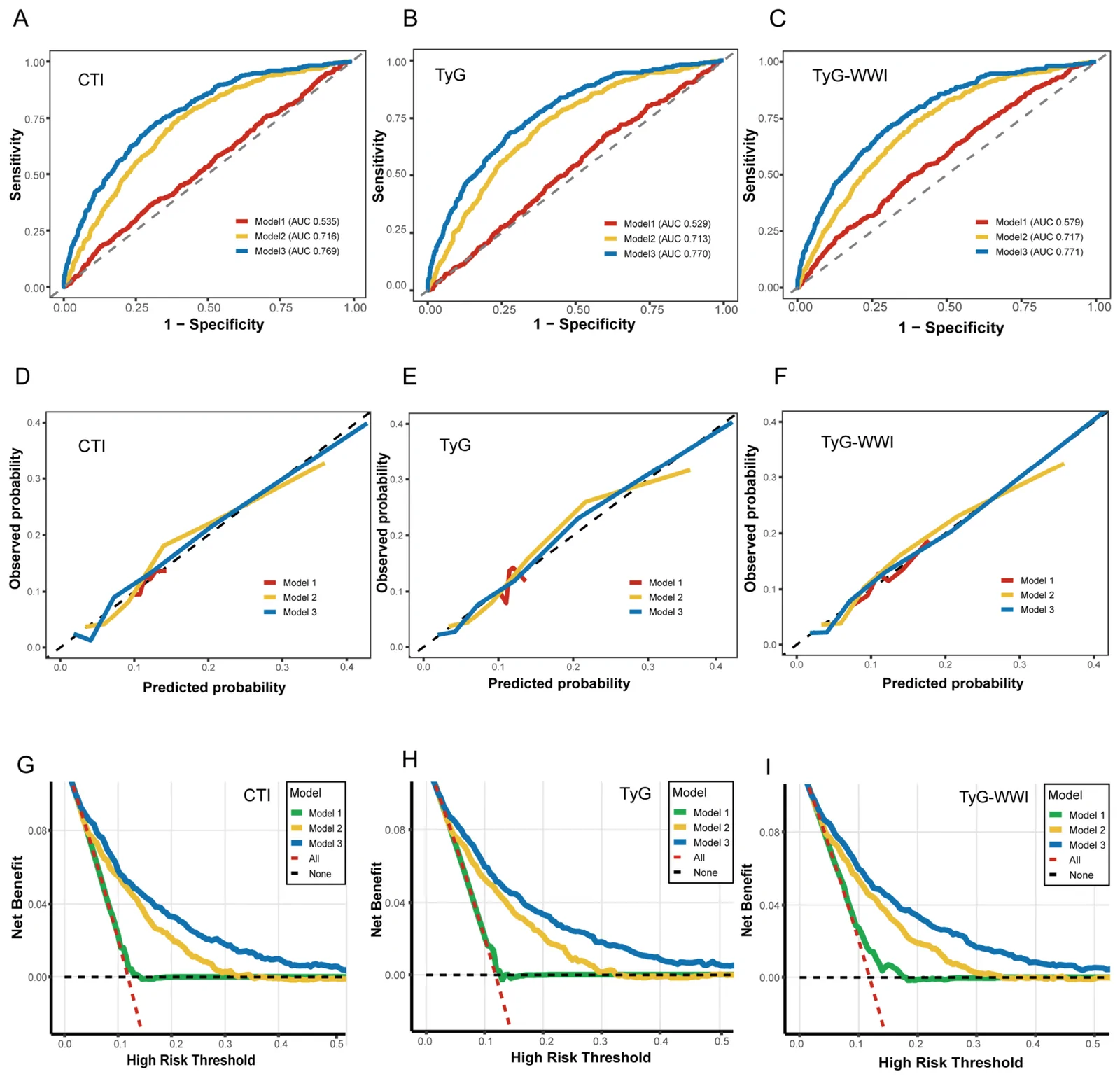
Comprehensive evaluation of novel insulin resistance indices for cardiovascular disease risk prediction in prediabetes patients. (**A**–**C**): Discriminatory performance (ROC curves) of (**A**) CTI, (**B**) TyG, and (**C**) TyG-WWI. (**D**–**F**): Calibration of the prediction models (bootstrap-adjusted). The dashed line represents perfect agreement. (**G**–**I**): Clinical utility (decision curve analysis) of models incorporating (**G**) CTI, (**H**) TyG, and (**I**) TyG-WWI.

This study evaluated the predictive performance of three novel IR indices—CTI, TyG, and TyG-WWI—for CVD risk in middle-aged and older adults with prediabetes. The results are summarized in Figure 3 and Table 3. All three indices demonstrated significant predictive ability for CVD in this population. ROC analysis revealed that after full adjustment for confounders (Model 3), the predictive accuracy of each index was substantially improved compared with the baseline model (Model 1). Specifically, the AUC values in Model 3 increased to 0.769 for CTI, 0.770 for TyG, and 0.771 for TyG-WWI, compared with corresponding values of 0.535, 0.529, and 0.579 in Model 1, indicating markedly enhanced discriminative capability. The three indices showed broadly comparable discriminative ability. The DeLong test confirmed that the differences in AUC between the fully adjusted model and both the baseline and partially adjusted models were statistically significant (all *p* < 0.05). Risk reclassification analysis showed significant improvements in both NRI and IDI for the fully adjusted model compared with the baseline model (all *p* < 0.001; Table 3). Quantitative calibration metrics demonstrated excellent model performance (Supplementary Table S5). Calibration curves indicated good agreement between predicted probabilities and observed event rates in the fully adjusted model. Furthermore, DCA demonstrated that the fully adjusted model provided higher net clinical benefit across a threshold probability range of 0–0.5 compared with the baseline model. Risk reclassification analysis (Supplementary Figure S2 and Table S4) showed that TyG-WWI provided a significant incremental value compared with the baseline clinical model.

### 3.5. Subgroup and Sensitivity Analyses

**Figure 4:**
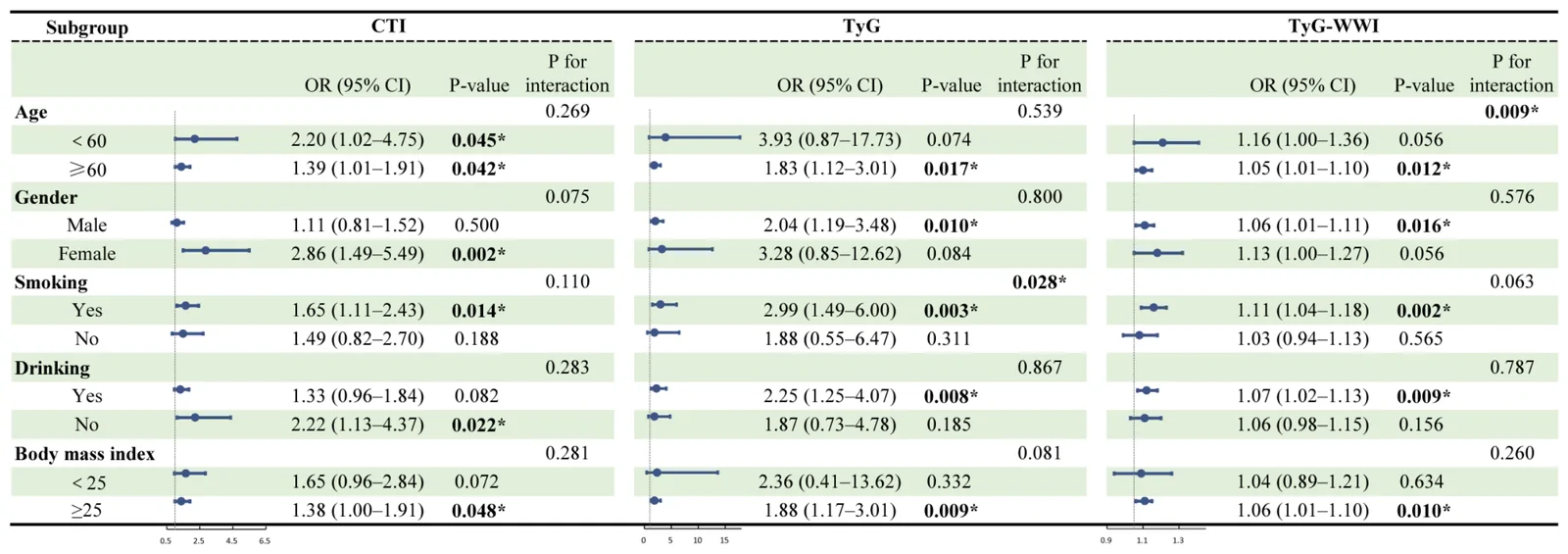
Subgroup analysis of the association between novel insulin resistance indices and CVD in prediabetic participants. CTI, C-reactive protein–triglyceride–glucose index; TyG, triglyceride–glucose index; TyG-WWI, triglyceride–glucose weight-adjusted waist index; model was adjusted for age, sex, marital status, education, Hypertension, Smoking, TC, TG, SBP, DBP, BUN, Uric Acid, Creatinine, BMI, WC, CRP, FBG, Height, and Weight. * *p* < 0.05.

To further explore the associations between the three novel IR indices (CTI, TyG, and TyG-WWI) and CVD, we conducted stratified analyses and interaction tests based on potential risk factors, with results presented in Figure 4. In the fully adjusted models, all three indices maintained positive associations with CVD, consistent with the primary findings. For CTI, significant positive correlations were observed in females, current smokers, non-drinkers, and individuals with obesity (BMI ≥ 25 kg/m^2^) (all *p* < 0.05). However, no significant interaction effects were detected for CTI across age, sex, smoking status, alcohol consumption, or BMI categories (all *p* for interaction > 0.05). The association between TyG and CVD demonstrated a significant interaction with smoking status (*p* for interaction = 0.028). The risk estimate was notably higher among current smokers (OR = 2.99, 95% CI: 1.49–6.00) compared with non-smokers (OR = 1.88, 95% CI: 0.55–6.47). No other significant interaction effects were observed for TyG across the remaining stratification factors. For TyG-WWI, a significant modifying effect was identified for age (*p* for interaction = 0.009), with a significant risk estimate observed in participants aged ≥60 years (OR = 1.05, 95% CI: 1.01–1.10). No other stratification factors showed significant interaction effects with TyG-WWI. Notably, after comprehensive adjustment for confounders, both TyG and TyG-WWI demonstrated significant positive associations with CVD risk in males, current smokers, drinkers, and individuals with obesity (BMI ≥ 25 kg/m^2^) (all *p* < 0.05).

### 3.6. The Mediating Role of CTI and TyG in the Effect of WWI on CVD in Prediabetic Patients

To further explore whether WWI is associated with CVD through IR and inflammation pathways, we conducted an exploratory mediation analysis. Novel IR indicators (CTI and TyG) were incorporated as mediators to decompose the total effect of WWI on CVD into direct and indirect effects. In the fully adjusted model (Model 2), as illustrated in Figure S3, WWI exhibited a significant positive total effect on CVD (all *p*-values for total effects < 0.05). Furthermore, significant mediating effects of CTI and TyG were observed (all *p*-values < 0.05). After covariate adjustment, the mediation proportions ranged from 22.13% to 26.30%, with CTI demonstrating the highest mediation proportion (26.30%). No significant direct effect of WWI on CVD was detected (*p* > 0.05).

### 3.7. External Validation in the CHARLS Dataset

Supplementary Table S6 presents the baseline clinical characteristics of 3764 participants aged 45 years or older with prediabetes from the CHARLS cohort, among whom 491 had CVD. Consistent with findings from NHANES, the CVD group was significantly older, had a lower proportion of married individuals, and exhibited elevated levels of TG, WWI, and all three novel IR indices (TyG, TyG-WWI, and CTI) (all *p* < 0.05). To further validate the robustness of the observed associations, Participants were categorized into three subgroups according to tertiles of each IR index (CTI, TyG, and TyG-WWI). The prevalence of CVD was calculated across these subgroups. The results show that the prevalence of CVD increased with the increase in CTI, TyG and TyG-WWI, as shown in Figure S4. Specifically, participants in T3 of each index showed significantly higher CVD prevalence compared with those in T1 (all *p* < 0.05). In addition, ROC analysis showed that three indices show broadly comparable discriminative performance (Figure S5). Calibration analysis indicated good model calibration (Table S7). These findings collectively confirm that the positive associations between the three novel IR indices and CVD remain stable in the independent CHARLS cohort.

## 4. Discussion

This study represents the first comprehensive investigation, based on two nationally representative cohorts—NHANES and CHARLS—to systematically evaluate the cross-sectional associations between three novel IR indices (CTI, TyG, and TyG-WWI) and prevalent CVD in middle-aged and older adults with prediabetes. Multiple machine learning algorithms were employed to identify the most critical and relevant covariates. Multivariable-adjusted logistic regression and restricted cubic spline analyses consistently demonstrated a significant linear increase in CVD risk with rising levels of CTI, TyG, and TyG-WWI. ROC analysis revealed that all three indices demonstrated significant and broadly comparable discriminative ability for prevalent CVD. Calibration curves indicated strong agreement between predicted probabilities and observed outcomes, while DCA confirmed the net clinical benefit of these indices across a threshold probability range of 0–0.5. Subgroup analyses provided further insights: the association between TyG-WWI and CVD was particularly pronounced in older adults, whereas TyG demonstrated a stronger risk effect among current smokers. Furthermore, external validation in the independent CHARLS cohort confirmed that the positive associations between all three IR indices (CTI, TyG, and TyG-WWI) and CVD prevalence remained robust. These results suggest that IR is significantly associated with elevated odds of prevalent CVD in prediabetes.

Several studies have examined the association between IR and CVD, primarily focusing on the TyG index without comparing it to CTI or TyG combined with WWI in prediabetic populations. For example, a meta-analysis by J He et al. found that IR significantly increases CVD risk [28]. A longitudinal cohort study in Sweden also showed a strong link between IR and elevated CVD incidence [29]. Additionally, a study with 23,103 participants indicated that higher TyG index levels were associated with increased CVD mortality [30]. Unlike previous research, our study specifically targets prediabetic individuals, a group at higher cardiovascular risk. Our findings demonstrate that all three novel IR indices (CTI, TyG, and TyG-WWI) are significantly linked to increased odds of prevalent CVD, aligning with and extending existing evidence. Mechanistically, IR contributes to cardiovascular issues through hyperinsulinemia-induced pathways, such as accelerated atherosclerosis and enhanced sympathetic nervous system activity [31,32]. Importantly, our study shows that IR can drive hyperinsulinemia even in normoglycemic prediabetes, underscoring the need for early intervention in this high-risk population.

While BMI indicates general obesity, WC and WWI reflect central obesity. Previous studies have shown that elevated WC, WWI, and BMI are independently linked to increased CVD risk [33,34]. Consistent with these findings, our study found higher levels of WC, WWI, and BMI in the CVD subgroup. The risk associated with obesity depends not only on total fat mass but also on fat distribution patterns. Visceral fat accumulation is strongly correlated with IR [35], and their interaction is a key driver of obesity-related complications [36]. The TyG index is a reliable marker for assessing IR [37], which is crucial in the pathophysiology of obesity. Therefore, combining TyG with obesity indices enhances disease risk evaluation [38]. Our study is the first to show that in a prediabetic population, increasing levels of TyG-WWI—a combined index of TyG and WWI—are associated with a linear rise in the odds of prevalent CVD. Notably, our multivariable-adjusted models indicated that participants in the highest tertile of CTI, TyG, and TyG-WWI had approximately twofold higher odds of prevalent CVD (ORs ranging from 1.80 to 1.97). This finding aligns with various meta-analyses and studies indicating that IR, assessed through TyG and CTI, contributes to increased CVD risk [13,28,39]. Thus, IR may be a fundamental mechanism linking abnormal fat metabolism to prevalent CVD in obese individuals with prediabetes.

This study evaluated the discriminative performance of three IR indices—CTI, TyG, and TyG-WWI—for prevalent CVD in middle-aged and older adults with prediabetes through ROC analysis. All three indices demonstrated significant and broadly comparable predictive value (AUC: 0.769–0.771). This finding aligns with previous studies highlighting the utility of TyG-based indices and CTI in assessing cardiovascular risk [13]. The superior performance of TyG-WWI may be due to its integration of two pathological dimensions: TyG captures dysregulation of glucose and lipid metabolism, while WWI reflects central obesity. Research has shown that visceral fat accumulation contributes to cardiovascular issues through mechanisms like chronic inflammation and endothelial dysfunction [40]. Our subgroup analysis revealed that three indices show broadly comparable discriminative performance in the elderly, who often present with central obesity and metabolic abnormalities. While the conventional TyG index effectively reflects glucolipid metabolic disorders related to IR, it does not account for body fat distribution as an independent risk factor. The integration of TyG and WWI enables simultaneous tracking of metabolic dysregulation and central obesity progression, supporting serial risk reassessment in clinical practice. Thus, given their broadly comparable performance, three indices may serve as useful tools for CVD risk stratification in prediabetes, a finding that underscores the key role of comprehensive treatment.

Studies have shown that impaired insulin signaling in diabetes leads to downregulation of GLUT4 expression in adipocytes and muscle cells, reduced insulin receptor numbers, excessive serine phosphorylation of IRS-1/2, and decreased PI3K-Akt activity, all of which suppress glucose uptake [41]. These molecular changes exacerbate IR and increase obesity risk. Additionally, the accumulation of lipid metabolites like diacylglycerol and ceramide activates PKCε/PKCθ pathways, creating a “lipotoxic” vicious cycle that complicates insulin sensitivity regulation [42]. Dysfunctional adipose tissue induces macrophage infiltration and releases pro-inflammatory cytokines (TNF-α, IL-6), activating the NF-κB/JNK pathway and exacerbating IR and endothelial dysfunction [43,44]. Oxidative stress, driven by mitochondrial dysfunction, further promotes vascular damage through ROS-mediated lipid peroxidation [45,46]. Therefore, this study highlights the importance of using composite indices that integrate both obesity and IR parameters to predict CVD risk in prediabetic individuals. These findings suggest that managing prediabetes should adopt an integrated approach addressing both obesity and IR to effectively reduce CVD risk.

Our findings should be interpreted within the broader prediabetes multimorbidity spectrum, which encompasses cognitive decline, frailty and CKD, and HFpEF [47,48,49]. The observed interaction between TyG-WWI and age may reflect metabolic–frailty convergence, consistent with the benefits of metformin in prefrail hypertensive older adults [50]. Machine learning–assisted risk stratification [51] could inform integrated interventions—including lifestyle modification and prediabetes reversal [52,53], Mediterranean dietary patterns [54], and pharmacological strategies [55,56]—to address the full spectrum of complications. Future studies should integrate cardiac structural and functional assessments, frailty evaluation, and dietary or pharmacological interventions to fully elucidate the IR–CVD axis and develop comprehensive prevention strategies.

This study is the first comprehensive investigation integrating two nationally representative cohorts—NHANES and CHARLS—to examine the association between IR and CVD risk in middle-aged and older adults with prediabetes. By using multiple machine learning algorithms to identify key confounding variables, our analysis provides strong evidence for developing CVD prevention strategies and establishes a theoretical foundation for precise early intervention. However, several limitations should be noted. The CHARLS database lacks detailed age data for individuals under 45, limiting in-depth analysis of IR and CVD in this younger group. Additionally, the cross-sectional design limits the causal inference of the observed association: since the direction of time cannot be determined, mediation analysis cannot exclude reverse causality. CVD outcomes, physical activity and dietary intake data were based on self-report, which may be affected by recall bias and measurement error. Future research should establish prospective cohorts to clarify temporal relationships and strengthen causal conclusions. Moreover, subsequent studies should include comprehensive assessments of lifestyle factors, metabolic status, and obesity management to develop tailored intervention strategies for CVD prevention in prediabetic populations.

## 5. Conclusions

The findings of this study demonstrate that IR, as assessed by CTI, TyG, and TyG-WWI, is independently associated with elevated odds of prevalent CVD in middle-aged and older adults with prediabetes. These results underscore the importance of integrating both central obesity and IR status into risk assessment and intervention strategies to reduce CVD burden in this high-risk population.

## Figures and Tables

**Table 1:** Characteristics of the study population by cardiovascular disease status.

Characteristics	Non-CVD (*n* = 2680, 85.9%)	CVD (*n* = 441, 14.1%)	*p*-Value
Age (years)	57.00 (50.00, 66.00)	69.00 (60.00, 77.00)	<0.001
Sex			0.003
males	8,882,119 (51%)	1,410,346 (61%)	
females	8,421,835 (49%)	912,668 (39%)	
Married, *n* (%)	12,500,872 (72%)	1,516,646 (65%)	0.029
Education level, %			<0.001
Below high school	3,301,227 (19%)	690,407 (30%)	
High school or GED	4,496,284 (26%)	562,649 (24%)	
Beyond high school	9,506,443 (55%)	1,069,959 (46%)	
Smoking group			<0.001
Never smoker	8,093,063 (47%)	799,679 (34%)	
Former smoker	6,022,463 (35%)	1,067,198 (46%)	
Current smoker	3,188,428 (18%)	456,138 (20%)	
Alcohol consumption			0.700
Non-drinker	4,422,916 (26%)	548,021 (24%)	
1–5 drinks/month	8,966,911 (52%)	1,261,546 (54%)	
6–10 drinks/month	848,075 (4.9%)	83,351 (3.6%)	
10+ drinks/month	3,066,052 (18%)	430,096 (19%)	
BMI group			0.130
Underweight	172,242 (1.0%)	53,932 (2.3%)	
Normal	3,819,342 (22%)	589,321 (25%)	
Overweight	6,893,067 (40%)	862,367 (37%)	
Obese	6,419,302 (37%)	817,393 (35%)	
Abdominal obesity-yes	11,042,367 (64%)	1,461,965 (63%)	0.700
Hypertension			<0.001
Yes	8,479,129 (49%)	1,763,198 (76%)	
No	8,824,825 (51%)	559,816 (24%)	
SBP (mmHg)	125.00 (115.33, 137.33)	125.33 (113.33, 144.67)	0.300
DBP (mmHg)	72.00 (66.00, 79.00)	68.00 (60.00, 75.00)	<0.001
Height (cm)	169.00 (161.20, 177.10)	168.10 (160.90, 177.40)	0.700
Weight (kg)	81.70 (69.90, 95.30)	80.70 (67.80, 93.10)	0.200
Uric acid (umol/L)	339.00 (285.50, 386.60)	356.90 (297.40, 428.30)	<0.001
Triglycerides (mmol/L)	1.33 (0.95, 1.94)	1.43 (1.06, 1.98)	0.045
Total Cholesterol (mmol/L)	5.43 (4.78, 6.10)	4.86 (4.22, 5.64)	<0.001
Creatinine (umol/L)	79.56 (66.30, 88.40)	85.75 (70.70, 99.01)	<0.001
FBG (mg/dL)	104.20 (100.40, 109.50)	105.00 (100.80, 111.00)	0.068
HbA1c (%)	5.60 (5.30, 5.80)	5.60 (5.40, 5.80)	0.075
BUN (mmol/L)	5.00 (3.93, 6.07)	5.36 (4.28, 7.10)	<0.001
CRP (mg/dL)	0.22 (0.10, 0.49)	0.24 (0.10, 0.64)	0.150
BMI (kg/m^2^)	28.42 (25.29, 32.11)	28.04 (24.66, 31.70)	0.120
WC (cm)	100.50 (91.80, 109.60)	102.50 (94.00, 111.20)	0.094
WWI	11.08 (10.67, 11.56)	11.32 (10.97, 11.85)	<0.001
TyG	8.72 (8.38, 9.09)	8.81 (8.51, 9.13)	0.021
TyG-WWI	97.37 (91.01, 104.02)	100.38 (94.27, 107.32)	<0.001
CTI	9.10 (8.54, 9.62)	9.19 (8.74, 9.70)	0.016

CVD, cardiovascular disease; Non-CVD, no cardiovascular disease; BMI, body mass index; SBP, systolic blood pressure; DBP, diastolic blood pressure; FBG, fasting blood glucose; HbA1c, hemoglobin A1c; BUN, blood urea nitrogen; CRP, C-reactive protein; WC, waist circumference; WWI, weight-adjusted waist index; TyG, triglyceride–glucose index; CTI, C-reactive protein–triglyceride–glucose index. Mean (standard deviation, SD) of normal continuous variables; median (interquartile range, IQRs) of skewed continuous variables; weighted frequency and percentage of categorical variables (*n* [%]). Continuous variables were analyzed by Mann–Whitney U test or *t*-test for complex survey samples; categorical variables were evaluated by Rao–Scott-corrected chi-square test. *p* < 0.05.

**Table 2:** Logistic regression analyses of the association of tertiles of IR-indices (CTI, TyG, TyG-WWI) with CVD.

	Model 1		Model 2		Model 3	
	OR (95% CI)	*p*	OR (95% CI)	*p*	OR (95% CI)	*p*
CTI						
T1	Ref		Ref		Ref	
T2	1.36 (0.99, 1.86)	0.055	1.27 (0.90, 1.80)	0.165	1.31 (0.92, 1.87)	0.136
T3	1.50 (1.10, 2.06)	0.012 *	1.63 (1.17, 2.26)	0.004 *	1.80 (1.20, 2.71)	0.005 *
TyG						
T1	Ref		Ref		Ref	
T2	1.69 (1.24, 2.30)	0.001 *	1.69 (1.23, 2.33)	0.002 *	1.89 (1.34, 2.66)	<0.001 *
T3	1.51 (1.10, 2.08)	0.011 *	1.62 (1.18, 2.22)	0.003 *	1.95 (1.23, 3.09)	0.005 *
TyG-WWI						
T1	Ref		Ref		Ref	
T2	1.57 (1.19, 2.09)	0.002 *	1.33 (0.98, 1.80)	0.070	1.43 (0.98, 2.08)	0.062
T3	2.18 (1.64, 2.89)	<0.001 *	1.66 (1.24, 2.23)	0.001 *	1.97 (1.20, 3.23)	0.008 *

OR, Odds ratio; 95% CI, 95% confidence interval; CVD, cardiovascular disease; WWI, weight-adjusted waist index; TyG, triglyceride–glucose index; CTI, C-reactive protein–triglyceride–glucose index. Model 1 was unadjusted. Model 2 was adjusted for age, sex, marital status, and education. Model 3 was further adjusted for Hypertension, Smoking, TC, TG, SBP, DBP, BUN, Uric Acid, Creatinine, BMI, WC, CRP, FBG, Height, Weight. * *p* < 0.05.

**Table 3:** Incremental predictive value of CTI, TyG, and TyG-WWI for cardiovascular disease: NRI and IDI analyses across nested models.

Variables	CTI		TyG		TyG-WWI	
	95% CI	*p*-Value	95% CI	*p*-Value	95% CI	*p*-Value
NRI						
Model 1 vs. Model 2	0.361 (0.310, 0.419)	<0.001	0.377 (0.323, 0.431)	<0.001	0.333 (0.277, 0.392)	<0.001
Model 1 vs. Model 3	0.451 (0.392, 0.505)	<0.001	0.457 (0.400, 0.513)	<0.001	0.439 (0.377, 0.500)	<0.001
Model 2 vs. Model 3	0.193 (0.126, 0.266)	<0.001	0.206 (0.138, 0.281)	<0.001	0.210 (0.146, 0.288)	<0.001
IDI						
Model 1 vs. Model 2	0.290 (0.217, 0.334)	<0.001	0.286 (0.219, 0.333)	<0.001	0.249 (0.177, 0.293)	<0.001
Model 1 vs. Model 3	0.392 (0.327, 0.444)	<0.001	0.399 (0.329, 0.449)	<0.001	0.374 (0.303, 0.421)	<0.001
Model 2 vs. Model 3	0.177 (0.132, 0.247)	<0.001	0.193 (0.142, 0.259)	<0.001	0.194 (0.148, 0.264)	<0.001

CTI, C-reactive protein–triglyceride–glucose index; TyG, triglyceride–glucose index; TyG-WWI, triglyceride–glucose weight-adjusted waist index; NRI, net reclassification improvement; IDI, integrated discrimination improvement; Model 1 was unadjusted. Model 2 was adjusted for age, sex, marital status, and education. Model 3 was further adjusted for Hypertension, Smoking, TC, TG, SBP, DBP, BUN, Uric Acid, Creatinine, BMI, WC, CRP, FBG, Height, Weight. *p* < 0.001.

## Data Availability

The data included in the work is accessible on https://www.cdc.gov/nchs/nhanes/index.html, (accessed on 13 February 2026) and https://charls.pku.edu.cn/en/index.htm, (accessed on 14 February 2026).

## Supplementary Materials

The following supporting information can be downloaded at: https://www.mdpi.com/article/10.3390/healthcare14162617/s1 [57,58,59].

## Abbreviations

CVD	cardiovascular disease
TyG	Triglyceride–glucose index
CTI	C-reactive protein–triglyceride–glucose index
WWI	weight-adjusted waist index
BMI	body mass index
WC	waist circumference
IR	Insulin resistance
SBP	systolic blood pressure
DBP	diastolic blood pressure
FPG	fasting plasma glucose
HbA1c	hemoglobin A1c
TC	total cholesterol
TG	triglycerides
UA	uric acid
BUN	blood urea nitrogen
CRP	C-reactive protein
