# Supplementary material for: Insulin Resistance Indices and Cardiovascular Disease Risk in Middle-Aged and Older Adults with Prediabetes: A Machine Learning-Assisted Predictive Modeling Study
Source: Healthcare (Basel). 2026 Aug 19;14(16):2617. doi: 10.3390/healthcare14162617 (PMC13512461; doi:10.3390/healthcare14162617)
Supplement: Supplementary file 1 [file healthcare-14-02617-s001.zip › healthcare-4325318-supplementary.pdf]

Supplementary Material 1

Appendix to "Insulin Resistance Indices and Cardiovascular Disease Risk in Middle-Aged and Older Adults with Prediabetes: A Machine Learning-Assisted Predictive Modeling Study"

Contents

Section 1: Participant selection flow---3

Figure S1. The flowchart in NHANES describes the choice of participants----------------------4

Section 2: Feature selection of machine learning --- 4

Table S1. The top 10 most significant features were selected by machine learning methods---5

Section 3: Sensitivity analysis -- 5

Table S2. Sensitivity analysis of the associations between insulin resistance indices and cardiovascular disease --5

Table S3. Sensitivity analysis of the associations between insulin resistance indices and cardiovascular disease after excluding height and weight from Main model---------------------6

Section 4: Model evaluation -- 8

Figure S2. Weighted receiver operating characteristic analysis of nested models for predicting cardiovascular disease in prediabetic adults. ---8

Table S4. Continuous NRI and IDI comparing TyG-WWI and TyG against the clinical baseline model --8

Table S5. Quantitative calibration metrics for CTI, TyG, and TyG-WWI in Model 3 -----------9

Section 5: Mediation analysis -- 9

Figure S3. Mediation analysis of CTI and TyG in the impact of WWI on CVD prediabetic patients --9

Section 6: Analysis of the CHARLS dataset --- 9

Table S6. Characteristics of the study population by cognition status ----------------------------9

Figure S4. Prevalence of CVD grouped by tertiles of CTI, TyG, and TyG-WWI ------------11

Figure S5. Receiver operating characteristic curves for cardiovascular disease prediction in prediabetic patients from the CHARLS database ---11

Table S7. Quantitative calibration metrics for CTI, TyG, and TyG-WWI in the fully adjusted model (CHARLS) ---11

Section 1: Participant selection flow

Study population and selection criteria

This analysis utilized data from the China Health and Retirement Longitudinal Study (2011). Initially, 4572 participants aged 45 years or older with prediabetes were included. Prediabetes was defined according to the American Diabetes Association criteria [1], characterized by either a fasting plasma glucose level between 100 mg/dL (5.6 mmol/L) and 125 mg/dL (6.9 mmol/L), or a glycated hemoglobin (HbA1c) level ranging from 5.7% to 6.4%.

Exclusion criteria were applied as follows: (1) Missing key data, including demographic and anthropometric measures (age, sex, height, weight, waist circumference) or essential biochemical or laboratory parameters (fasting glucose, HbA1c, C-reactive protein, triglycerides), leading to the exclusion of 800 individuals; (2) Incomplete cardiovascular disease information, defined as lack of responses to relevant questionnaires (encompassing heart disease, angina, coronary heart disease, heart failure, other heart conditions) or self-reported history of stroke, resulting in the exclusion of 8 participants. After applying these criteria, a total of 3764 participants were included for subsequent statistical analyses.

Screening and evaluation of cardiovascular disease

In the CHARLS study, cardiovascular disease (CVD) was defined as a composite of cardiac conditions (including coronary heart disease, heart failure, and related disorders) and cerebrovascular diseases (such as stroke). CVD status was determined based on affirmative responses to the self-reported question: “Has a doctor ever diagnosed you with heart disease, angina, coronary heart disease, heart failure, or other heart problems?” Participants who reported a prior physician diagnosis of myocardial infarction, coronary heart disease, heart failure, or stroke were classified as having CVD [2, 3].

Data collection and definitions

A comprehensive set of covariates was systematically extracted from the CHARLS database. These included sociodemographic factors (sex, age, educational level, and marital status), lifestyle behaviors (smoking status and alcohol consumption), and anthropometric measurements (body weight, height, waist circumference, body mass index, and systolic and diastolic blood pressure). Body mass index (BMI) was categorized as underweight (<18.5 kg/m^2), normal (18.5–24.9 kg/m^2), overweight (25.0–29.9 kg/m^2), or obese (≥ 30.0 kg/m^2). Abdominal obesity was defined as a waist circumference ≥ 102 cm for men and ≥ 88 cm for women. Hypertension was identified based on either a previous diagnosis or measured systolic blood pressure ≥ 140 mmHg or diastolic blood pressure ≥ 90 mmHg. Laboratory parameters included fasting blood glucose (FBG), glycated hemoglobin (HbA1c), triglycerides (TG), total cholesterol (TC), uric acid, serum creatinine, blood urea nitrogen (BUN), and C-reactive protein (CRP).

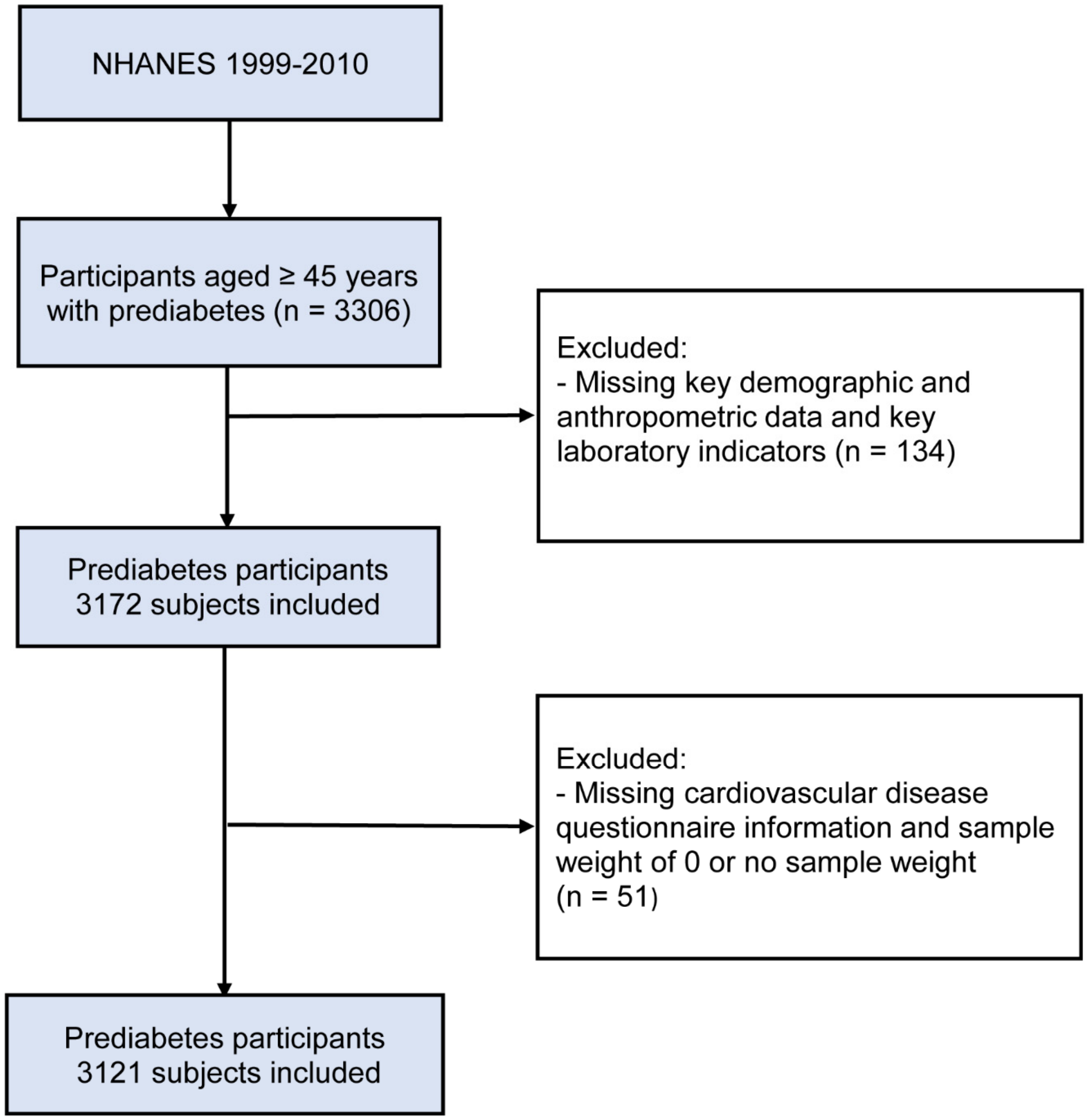

Figure S1. The flowchart in NHANES describes the choice of participants

Section 2: Feature selection of machine learning

Details of the machine learning

To optimize model performance while adjusting for confounding factors, four machine learning algorithms were employed to identify the core predictive variables. For support vector machine–recursive feature elimination (SVM-RFE), the caret package was used to perform recursive feature elimination, with feature ranking based on lrFuncs. The final model used a radial basis

function kernel (svmRadial), with the cost parameter C set to the default value of 1 in caret; five-fold cross-validation (rfeControl(method = "cv", number = 5)) was embedded in the recursive feature elimination procedure. For LASSO regression, an L1 penalty (alpha = 1) was applied, and the optimal regularization parameter λ was selected through 10-fold cross-validation. The lambda.min, which minimized the binomial deviance, was chosen as the final tuning parameter. Boruta was implemented based on random forest with 500 trees (ntree = 500), and variable importance was assessed by comparing each predictor with its shadow features. For XGBoost, the model was constructed within a gradient boosting framework. The number of boosting rounds (nrounds) was set to 50 or 100, and the maximum tree depth (max_depth) to 3 or 4, whereas the learning rate (eta) was fixed at 0.1, the subsample ratio at 0.8, and the column sampling ratio per tree (colsample_bytree) at 0.7. Five-fold repeated cross-validation (repeatedcv, 5 folds, 1 repeat) was used to determine the optimal parameter combination based on the maximum area under the receiver operating characteristic curve (AUC), and information gain was used to quantify variable importance. For each algorithm, the top ten predictors most strongly associated with cardiovascular disease were selected according to the respective variable importance criteria.

Table S1. The top 10 most significant features were selected by machine learning methods.

**Algorithms**	**Boruta**	**SVM-RFE**	**XGBoost**	**LASSO**
CVD	BMI, WC, Age, Sex, Height, Weight, TG, CRP, TyG, TyG-WWI	Age, Hypertension, TC, Smoke, SBP, BUN, Sex, Uric acid, Marital, Creatinine	Age, TC, Creatinine, SBP, DBP, Hypertension, TyG-WWI, BUN, Height, FBG	Hypertension, Sex, TC, Smoke, Marital, BUN, Age, TyG, TyG-WWI, Education
Model 3	Age, Hypertension, TC, Smoke, SBP, BUN, Sex, Uric acid, Marital, Creatinine, BMI, WC, Height, Weight, TG, CRP, DBP, FBG, Education			

Section 3: Sensitivity analysis

Table S2. Sensitivity analysis of the associations between insulin resistance indices and cardiovascular disease

		**OR (95% CI)**	***P***
**CTI**			
	**T1**	Ref	
	**T2**	1.31 (0.91, 1.90)	0.143

	**T3**	2.01 (1.25, 3.22)	**0.005***
**TyG**			
	**T1**	Ref	
	**T2**	1.87 (1.30, 2.68)	**0.001***
	**T3**	1.75 (1.06, 2.89)	**0.030***
**TyG-WWI**			
	**T1**	Ref	
	**T2**	1.35 (0.94, 1.95)	0.107
	**T3**	1.79 (1.08, 2.97)	**0.024***

OR, Odds ratio; 95% CI, 95% confidence interval; CVD, cardiovascular disease; WWI, weight-adjusted waist index; TyG, triglyceride glucose index; CTI, C-reactive protein–triglyceride–glucose index. Model was adjusted for age, sex, marital status, education, Hypertension, Smoking, TC, TG, SBP, DBP, BUN, Uric Acid, Creatinine, BMI, WC, CRP, FBG, Height, Weight, antihypertensive drugs, lipid-lowering drugs and hypoglycemic drugs. * P <0.05.

Table S3. Sensitivity analysis of the associations between insulin resistance indices and cardiovascular disease

		**Model 1**		**Model 2**		**Model 3**	
		**OR (95% CI)**	***P***	**OR (95% CI)**	***P***	**OR (95% CI)**	***P***
**CTI**							
	**T1**	Ref		Ref		Ref	
	**T2**	1.31 (0.92, 1.87)	0.136	1.32 (0.93, 1.88)	0.123	1.31 (0.94, 1.82)	0.120
	**T3**	1.80 (1.20, 2.71)	**0.005***	1.82 (1.21, 2.74)	**0.006***	1.76 (1.26, 2.46)	**0.001***
**TyG**							
	**T1**	Ref		Ref		Ref	

**T2**	1.89 (1.34, 2.66)	**<0.001***	1.90 (1.36, 2.66)	**<0.001***	1.87 (1.34, 2.60)	**<0.001***
**T3**	1.95 (1.23, 3.09)	**0.005***	1.97 (1.25, 3.09)	**0.005***	1.96 (1.43, 2.68)	**<0.001***
**TyG-WWI**						
**T1**	Ref		Ref		Ref	
**T2**	1.43 (0.98, 2.08)	0.062	1.39 (0.95, 2.05)	0.096	1.32 (0.96, 1.81)	0.089
**T3**	1.97 (1.20, 3.23)	**0.008***	1.85 (1.15, 2.96)	**0.013***	1.73 (1.28, 2.33)	**<0.001***

OR, Odds ratio; 95% CI, 95% confidence interval; CVD, cardiovascular disease; WWI, weight-adjusted waist index; TyG, triglyceride glucose index; CTI, C-reactive protein–triglyceride–glucose index. Model 1 was adjusted for age, sex, marital status, education, Hypertension, Smoking, TC, TG, SBP, DBP, BUN, Uric Acid, Creatinine, BMI, WC, CRP, FBG, Height, Weight, antihypertensive drugs, lipid-lowering drugs and hypoglycemic drugs. Model 2 was adjusted for age, sex, marital status, education, Hypertension, Smoking, TC, TG, SBP, DBP, BUN, Uric Acid, Creatinine, BMI, WC, CRP, FBG, antihypertensive drugs, lipid-lowering drugs and hypoglycemic drugs. Model 3 was adjusted for age, sex, marital status, education, Hypertension, Smoking, TC, SBP, DBP, BUN, Uric Acid, Creatinine, CRP, Height, antihypertensive drugs, lipid-lowering drugs and hypoglycemic drugs. * $P < 0.05$.

Section 4: Model evaluation

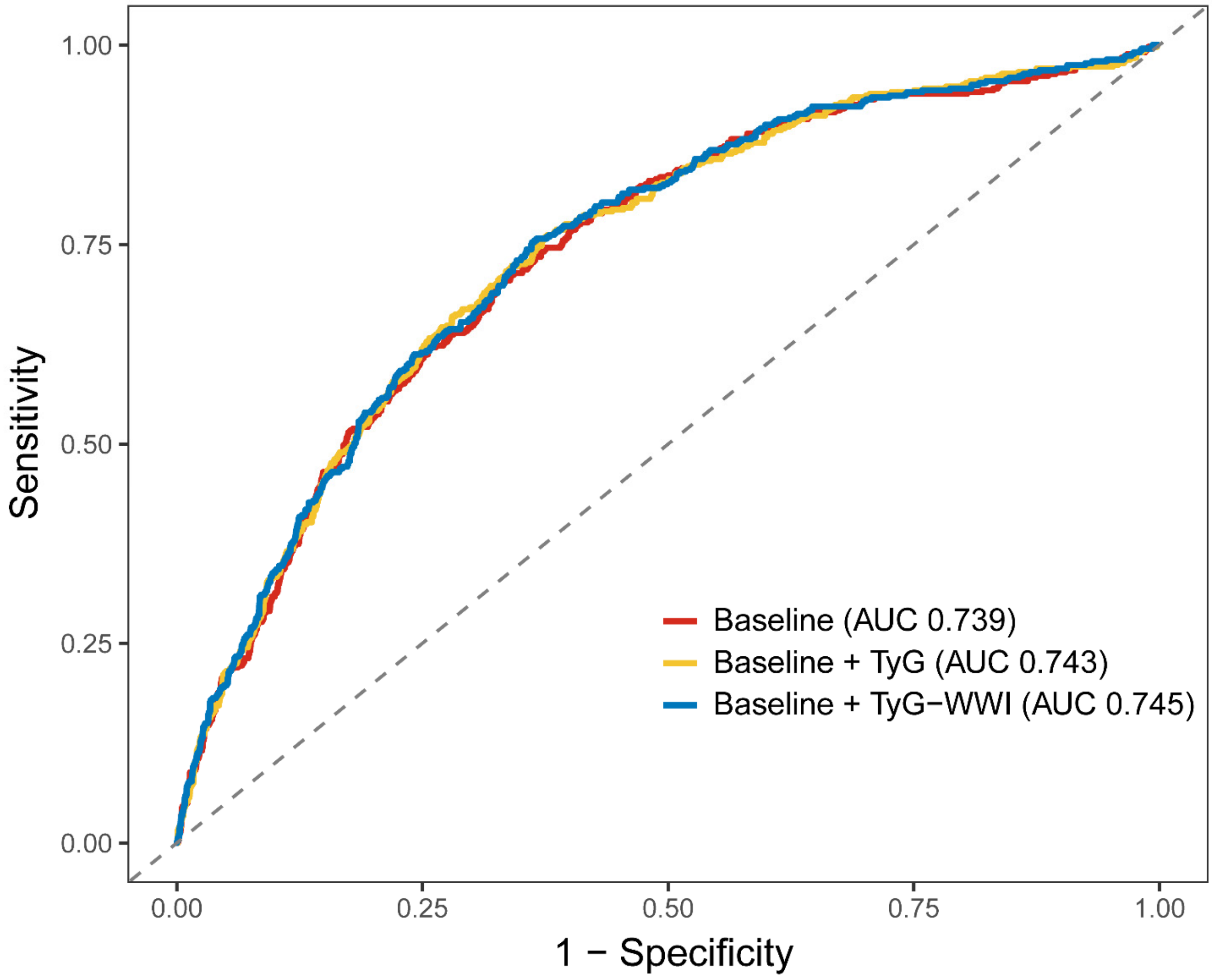

Figure S2. Weighted receiver operating characteristic analysis of nested models for predicting cardiovascular disease in prediabetic adults. CVD, cardiovascular disease; WWI, weight-adjusted waist index; TyG, triglyceride glucose index; CTI, C-reactive protein–triglyceride–glucose index.

Table S4. Continuous NRI and IDI comparing TyG-WWI and TyG against the clinical baseline model

Variables	NRI	*P* value	IDI	*P* value
Baseline vs +TyG	0.0222 (0.0036, 0.0649)	0.034	0.0058 (0.0037, 0.0105)	0.001
Baseline vs +TyG-WWI	0.0431 (0.0120, 0.0808)	0.004	0.0068 (-0.0005, 0.0098)	0.078
Baseline+TyG vs Baseline+TyG-WWI	0.0210 (-0.0202, 0.0436)	0.434	0.0010 (-0.0067, 0.0021)	0.33

TyG, triglyceride glucose index; TyG-WWI, triglyceride glucose-weight adjusted waist index; NRI, net reclassification improvement; IDI, integrated discrimination improvement.

Table S5. Quantitative calibration metrics for CTI, TyG, and TyG-WWI in Model 3

	Model	Calibration Slope	Calibration Intercept	Brier Score
CTI	Model 3	1.011	-0.039	0.088
TyG	Model 3	1.002	-0.058	0.088
TyG WWI	Model 3	1.003	-0.046	0.088

WWI, weight-adjusted waist index; TyG, triglyceride glucose index; CTI, C-reactive protein–triglyceride–glucose index.

Section 5: Mediation analysis

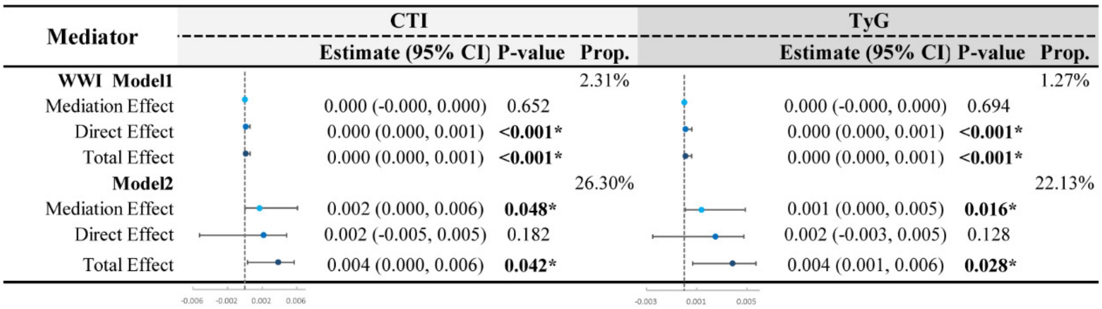

Figure S3. Mediation analysis of CTI and TyG in the impact of WWI on CVD prediabetic patients. CTI, C-reactive protein–triglyceride–glucose index; TyG, triglyceride glucose index; WWI, weight adjusted waist index. Model 1 was unadjusted. Model 2 was adjusted for age, sex, marital status, education, Hypertension, Smoking, TC, TG, SBP, DBP, BUN, Uric Acid, Creatinine, CRP, FBG, * P <0.05.

Section 6: Analysis of the CHARLS dataset

Table S6. Characteristics of the study population by cognition status

Characteristics	Non-CVD (n =3273, 87.0%)	CVD (n = 491, 13.0%)	P value
**Age (years)**	59.37 ± 9.26	62.10 ± 9.01	**<0.001**
**sex**			0.06
female	1734 (52.98)	283 (57.64)	
male	1539 (47.02)	208 (42.36)	
**marital status**			**<0.001**
no	412 (12.59)	94 (19.14)	
yes	2861 (87.41)	397 (80.86)	
**education**			**0.03**
College or above	43 (1.31)	10 (2.04)	
master's degree	929 (28.38)	136 (27.70)	
Middle school	1581 (48.30)	223 (45.42)	

No formal education	720 (22.00)	121 (24.64)	
**smoke**			**<0.001**
Current smoker	989 (30.22)	103 (20.98)	
ever smoke, don't known whether smoke now	8 (0.24)	3 (0.61)	
Former smoker	285 (8.71)	72 (14.66)	
Never	1991 (60.83)	313 (63.75)	
**drink**			**<0.001**
no	2149 (65.66)	381 (77.60)	
yes	1124 (34.34)	110 (22.40)	
**obesity**			**<0.001**
low weight	196 (5.99)	32 (6.52)	
normal	1677 (51.24)	195 (39.71)	
obesity	374 (11.43)	87 (17.72)	
over weight	1026 (31.35)	177 (36.05)	
**SBP (mmHg)**	131.16 ± 21.23	135.25 ± 22.19	**<0.001**
**DBP (mmHg)**	76.05 ± 11.91	77.43 ± 12.46	**0.02**
**Height (m)**	1.58 ± 0.09	1.58 ± 0.09	0.48
**Weight (kg)**	59.04 ± 11.33	61.40 ± 12.51	**<0.001**
**Uric acid (mg/dL)**	4.51 ± 1.26	4.57 ± 1.27	0.38
**Triglycerides (mg/dL)**	131.64 ± 86.70	141.76 ± 84.73	**0.01**
**Total Cholesterol (mg/dL)**	198.06 ± 38.97	198.72 ± 38.98	0.73
**Creatinine (mg/dL)**	0.78 ± 0.27	0.81 ± 0.24	**0.02**
**FBG (mg/dL)**	108.42 ± 7.81	108.60 ± 7.76	0.65
**HbA1c (%)**	5.21 ± 0.42	5.24 ± 0.41	0.19
**CRP (mg/L)**	2.74 ± 7.61	2.63 ± 5.74	0.71
**BMI (kg/m^2)**	23.62 ± 3.79	24.46 ± 4.24	**<0.001**
**WC (cm)**	84.61 ± 12.12	87.53 ± 12.83	**<0.001**
**WWI**	11.08 ± 1.29	11.25 ± 1.31	**<0.01**
**TyG**	8.71 ± 0.55	8.80 ± 0.52	**<0.001**
**TyG -WWI**	96.47 ± 13.25	99.08 ± 13.54	**<0.001**
**CTI**	4.78 ± 0.54	4.86 ± 0.54	**<0.01**

Values are reported as mean ± SD, number (percentage) or median (interquartile range). CVD, cardiovascular disease; Non-CVD, no cardiovascular disease; BMI, body mass index; SBP, systolic blood pressure; DBP, diastolic blood pressure; FBG, fasting blood glucose; HbA1c, hemoglobin A1c; CRP, C-reactive protein; WC, waist circumference; WWI, weight-adjusted waist index; TyG, triglyceride glucose index; CTI, C-reactive protein–triglyceride–glucose index. * P <0.05.

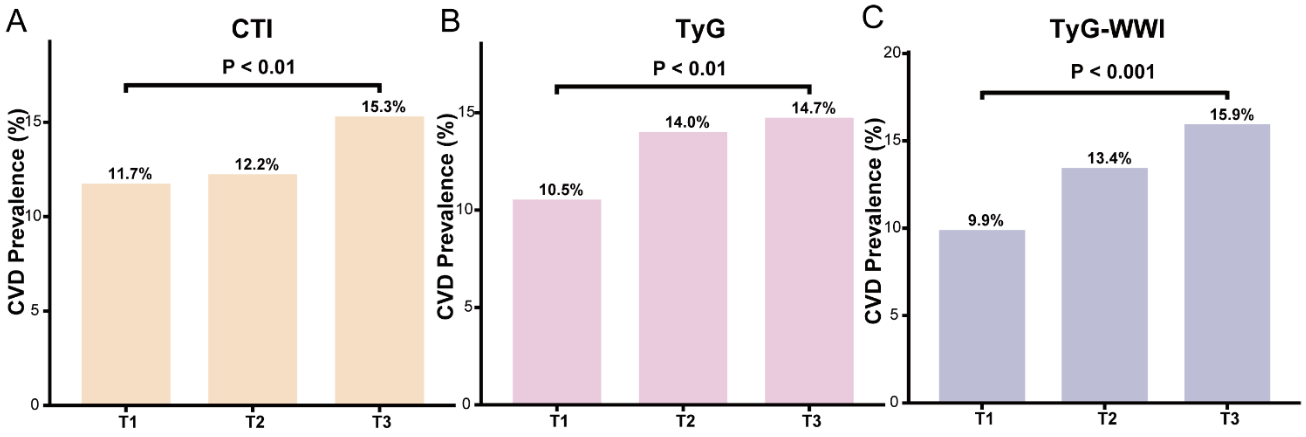

Figure S4. Prevalence of CVD grouped by tertiles of CTI, TyG, and TyG-WWI. CVD, cardiovascular disease; WWI, weight-adjusted waist index; TyG, triglyceride glucose index; CTI, C-reactive protein–triglyceride–glucose index.

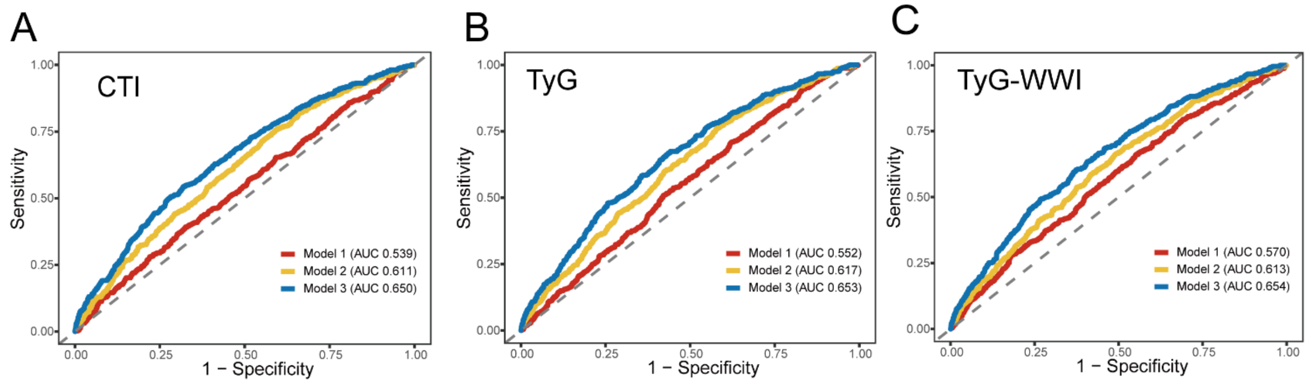

Figure S5. Receiver operating characteristic curves for cardiovascular disease prediction in prediabetic patients from the CHARLS database. (A) CTI, (B) TyG, and (C) TyG- WWI.

Table S7. Quantitative calibration metrics for CTI, TyG, and TyG-WWI in the fully adjusted model (CHARLS)

	Calibration Slope	Calibration Intercept	Brier Score
CTI	1.008	0.020	0.109
TyG	1.021	0.046	0.109
TyG WWI	1.055	0.106	0.109

WWI, weight-adjusted waist index; TyG, triglyceride glucose index; CTI, C-reactive protein–triglyceride–glucose index.

Reference

1. Care D. Classification and diagnosis of diabetes. *Diabetes Care*. 2015;38(Suppl 1):S8-S16.

2. Gao K, Cao L-F, Ma W-Z, et al. Association between sarcopenia and cardiovascular disease among middle-aged and older adults: findings from the China health and retirement longitudinal study. *EClinicalMedicine*. 2022;44

3. He D, Wang Z, Li J, et al. Changes in frailty and incident cardiovascular disease in three prospective cohorts. *European heart journal*. 2024;45(12):1058-1068.
